# Serum proteomic profiling in patients with advanced *Schistosoma japonicum*-induced hepatic fibrosis

**DOI:** 10.1186/s13071-021-04734-1

**Published:** 2021-05-01

**Authors:** Jing Huang, Xinguang Yin, Lifang Zhang, Ming Yao, Dahai Wei, Yiming Wu

**Affiliations:** 1 Institute of Hepatology, The Affiliated Hospital of Jiaxing University, Jiaxing, 314001 Zhejiang People’s Republic of China; 2 Institute of Hepatology, The First Hospital of Jiaxing, Jiaxing, 314001 Zhejiang People’s Republic of China; 3 Department of Clinical Medicine, Bengbu Medical College, Bengbu, 233030 Anhui People’s Republic of China; 4 Jiaxing Maternity and Child Health Care Hospital, Jiaxing, 314001 Zhejiang People’s Republic of China

**Keywords:** Quantitative proteomics, Schistosomiasis-induced hepatic fibrosis, *Schistosoma japonicum*, C1QA, CFD

## Abstract

**Background:**

*Schistosoma japonicum* is a parasitic flatworm that is the aetiological agent of human schistosomiasis, an important cause of hepatic fibrosis. Schistosomiasis-induced hepatic fibrosis is a consequence of the highly fibrogenic nature of egg-induced granulomatous lesions, which are the main pathogenic features of schistosomiasis. Although global awareness of the association between schistosomiasis-induced hepatic fibrosis and *S. japonicum* infection is increasing, little is known about the molecular differences associated with rapid progression to schistosomiasis in cirrhotic patients.

**Methods:**

We systematically used data-independent acquisition (DIA)-based liquid chromatography-mass spectrometry to identify differentially expressed proteins in serum samples from patients with advanced *S. japonicum*-induced hepatic fibrosis.

**Results:**

Our analysis identified 1144 proteins, among which 66 were differentially expressed between the healthy control group and the group of patients with advanced *S. japonicum*-induced hepatic fibrosis stage F2 (SHF-F2) and 214 were differentially expressed between the SHF-F2 and SHF-F4 groups (up- or downregulation of at least 1.5-fold in serum samples). The results also indicated that two selected proteins (C1QA and CFD) are potential biomarkers for distinguishing between patients with SHF-F2 and those with SHF-F4 due to *S. japonicum* infection.

**Conclusions:**

We provide here the first global proteomic profile of serum samples from patients with advanced *S. japonicum*-induced hepatic fibrosis. The proteins C1QA and CFD are potential diagnostic markers for patients with SHF-F2 and SHF-F4 due to *S. japonicum* infection, although further large-scale studies are needed. Our DIA-based quantitative proteomic analysis revealed molecular differences among individuals at different stages of advanced *S. japonicum*-induced hepatic fibrosis and may provide fundamental information for further detailed investigations.

**Graphic Abstract:**

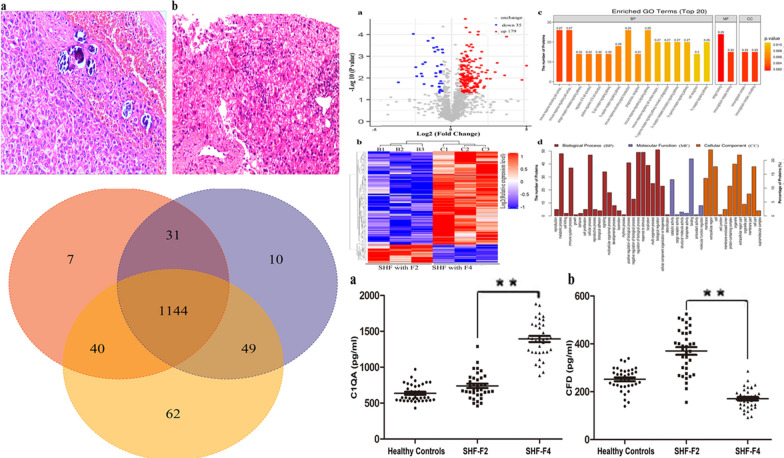

**Supplementary Information:**

The online version contains supplementary material available at 10.1186/s13071-021-04734-1.

## Background

Schistosomiasis, also called bilharzia, is the second most prevalent parasitic disease worldwide. It is caused by blood flukes (trematode worms) of the genus *Schistosoma* and annually over 229 million people worldwide are infected, leading to approximately 200,000 deaths [1, 2]. Schistosome infections, which are associated with chronic local inflammatory responses to schistosome eggs trapped in host tissues, can result in long-term, severe complications that include hepatic, intestinal, ureteral and bladder fibrosis and even bladder cancer and hepatocellular carcinoma (HCC) [3–5]. Epidemiological observations have clearly indicated that the main schistosomal species infecting humans are *Schistosoma japonicum*, *S. mansoni* and *S. haematobium* [5]. Among these, *S. japonicum* is the major species causing schistosomiasis in Southeast Asia, including China, and is associated with severe pathogenicity induced by the eggs of adult worms and subsequent schistosomiasis-induced hepatic fibrosis (SHF) that eventually develops into HCC, which is the main cause of schistosomiasis-related mortality [2, 5–7].

*Schistosoma japonicum* has a life-cycle that involves both an intermediate host (freshwater snails) and a definitive host (humans and other mammalians) [8, 9]. Eggs hatch upon contact with water, releasing miracidia, which penetrate the intermediate host, i.e. the snail, and develop into sporocysts [5]. Following infection of a human host, the schistosomula migrate to the portal-mesenteric vein system and are transported to the lungs* via* the right heart before reaching the left heart and entering the arterial circulation to travel to the liver through the hepatic portal system where they cause an inflammatory reaction with granuloma formation [10, 11]. The principal site of granuloma formation is around around the schistosome eggs trapped in host tissues, particularly those in the presinusoidal capillary venules of the liver [9]. Over time and/or with repeated or long-term infection, these granulomas, which develop at the sites of maximal egg accumulation, progress to severe and often irreversible SHF that disrupts hepatic blood flow, in turn obstructs portal venous flow and may lead to potentially life-threatening variceal bleeding [7, 12–14]. In addition, some data suggest that the granulomatous response and SHF in the liver continue to worsen and subsequently lead to the development of HCC, even after the administration of effective schistosomicides [15–18]. However, in some individuals, the egg-induced granulomatous response does not lead to severe SHF. Notably, the factors that can prevent this complication from developing remain poorly understood [3].

*Via* host–parasite interactions, schistosomes regulate host macromolecular synthesis by modifying the host transcription and translation machinery and forcing the host to meet the requirements of parasites during infection [19–21]. These requirements may lead to epigenetic modifications that are involved in various biological processes resulting from cellular immune responses and the generation of cytokine patterns elicited during the different stages of the parasite’s life-cycle throughout the course of infection [17, 18]. These processes subsequently lead to chronic immune response-based inflammation directed against schistosome eggs trapped in host tissues, gradually triggering the formation of periovular granulomas and eventually causing chronic SHF in infected individuals [22]. Accumulated data indicate that during schistosome infection, the host immune pattern gradually switches from a predominant T-helper 1 (Th1)-type response to a predominant Th2d response after egg deposition, which contributes to the development of hepatic fibrosis and portal hypertension through the synthesis of schistosome-specific immunoglobulin E (IgE) and the secretion of cytokines, such as interleukin (IL)-4, IL-5, IL-10 and IL-13 [23–25]. The development of SHF therefore appears to involve the interaction of many different factors, but the overall consequences depend on the degree of Th2 immune response activation [17, 25]. These observations indicate that marked differences exist in the molecular pathogenesis of *S. japonicum*-induced hepatic fibrosis among patients with SHF at different stages, although the precise molecular mechanism remains elusive.

Comparative proteomic approaches involving data-independent acquisition (DIA)-based liquid chromatography-tandem mass spectrometry (LC–MS/MS) are widely used to analyse host responses in animals and humans during parasite infections [26, 27]. The use of DIA-based quantitative proteomics to screen for diagnostic and prognostic protein biomarkers has also been reported [28, 29]. Clinical proteomic strategies provide an overall understanding of the host factors involved in parasite infection and provide insights into alterations in signalling pathways, thereby improving our understanding of parasite pathogenesis. Serum proteomic analysis is a valuable method for identifying protein biomarkers for application in the early recognition, diagnosis, monitoring and treatment of diseases, including parasite infections [27–30]. In the study reported here, to understand the molecular differences underlying host resistance to schistosomes, we used DIA-based quantitative proteomics to examine the differential expression of proteins in the pooled serum of patients with advanced *S. japonicum*-induced hepatic fibrosis. Our findings provide the first insights into the molecular differences between patients with advanced *S. japonicum*-induced hepatic fibrosis stage F2 (SHF-F2) and those with SHF stage F4 (SHF-F4) and may provide supply fundamental information for future research.

## Methods

### Patients and sample collection

The study population comprised 18 healthy controls and 36 eligible patients with advanced *S. japonica* infection. All patients and the gender-/age-matched healthy controls were enrolled between January 2015 and December 2019 from Jiaxing City, Zhejiang Province, China, which is endemic for *S. japonicum* infection. All patients were treated with praziquantel upon diagnosis of a *S. japonicum* infection during the 1960s to 1970s. SHF was staged from F0 to F4 according the METAVIR scoring system by two independent and experienced pathologists. Clinical laboratory parameters were measured and recorded using a Hitachi 7600 analyser (Hitachi High-Technologies Corp., Ibaraki, Japan) following the manufacturer’s instructions, on the same day as liver biopsy in the same laboratory [31]. A medical history, biochemical data and additional information on previous exposure to water involving schistosomal infection were also obtained, and a physical examination, sample collection and pathological analysis were performed. All data were recorded for each individual seen at The Affiliated Hospital of Jiaxing University, East China. Patients with the following conditions were excluded from the study: mixed liver disease; coinfection with other viruses, such as hepatitis A virus (HAV), HBV, HCV or HDV; decompensated liver disease; primary liver cancer or other malignant tumours; autoimmune or immunologically mediated disease; organ transplantation; or severe heart, liver or kidney function disorders. Relevant patient clinical information is present in Table 1.

**Table 1:** Baseline characteristics of patients enrolled in this study

Characteristic	Healthy control group (*n* = 18)	SHF stage F2 group^a^ (*n* = 18)	t-test (*n* = 18)^b^	P*	SHF stage F4 group^a^ (*n* = 18)	t-test (*n* = 18)^c^	*P*^*§*^
Gender (female/male)	9/9	8/10	N/A	N/A	10/8	N/A	N/A
Age (years)	74.83 ± 6.11	74.94 ± 6.73	− 0.052	0.959	75.00 ± 5.48	− 0.270	0.978
ALT (U/l)	13.72 ± 3.34	17.17 ± 5.32	− 2.328	0.026	35.89 ± 4.59^§^	− 11.312	< 0.001
AST (U/L)	18.44 ± 4.02	32.50 ± 7.34*	− 7.125	< 0.001	47.78 ± 9.52^§^	− 5.391	< 0.001
ALP (U/l)	29.11 ± 7.11	85.44 ± 15.89*	− 13.725	< 0.001	150.40 ± 42.11^§^	− 6.112	< 0.001
GGT (U/l)	21.39 ± 7.51	32.56 ± 19.25	− 2.292	0.032	146.39 ± 125.98^§^	− 3.789	0.001
PLT (10^9^ l^−1^)	220.61 ± 41.52	92.33 ± 17.99*	12.027	< 0.001	42.83 ± 19.78^§^	7.855	< 0.001
TB (umol/l)	6.34 ± 1.49	15.67 ± 4.03*	− 9.203	< 0.001	29.59 ± 14.95^§^	− 3.816	0.001
TBA (umol/l)	2.78 ± 1.40	3.88 ± 2.28	− 1.734	0.092	25.69 ± 14.50^§^	− 6.304	< 0.001
Albumin (g/l)	48.32 ± 3.23	47.59 ± 2.53	0.747	0.461	39.46 ± 2.83^§^	9.101	< 0.001
APRI	0.67 ± 0.18	2.73 ± 0.48*	− 16.921	< 0.001	10.15 ± 4.76^§^	− 6.584	< 0.001
PT (s)	N/A	12.89 ± 0.62	N/A	N/A	15.52 ± 1.27^§^	− 7.906	< 0.001
INR	N/A	1.00 ± 0.05	N/A	N/A	1.20 ± 0.10^§^	− 7.821	< 0.001
APTT (s)	N/A	37.27 ± 3.23	N/A	N/A	42.31 ± 3.09^§^	− 4.786	< 0.001
FIB (g/l)	N/A	2.95 ± 0.58	N/A	N/A	2.32 ± 0.43^§^	3.696	0.001

Values in table are present as the mean ± standard deviation

*ALP* Alkaline phosphatase, *ALT* alanine aminotransferase,* APRI* AST-to-PLT ratio index, *APTT* activated partial thromboplastin, * AST* aspartate aminotransferase,* FIB* fibrinogen content, * GGT* gamma-glutamyltranspeptidase,* INR* international normalized ratio,* N/A* information not available, * PLT* platelet count,* SHF* schistosomiasis-induced hepatic fibrosis,* PT* prothrombin time,* TB *total bilirubin,* TBA* total bile acid

*P value < 0.05 for comparisons between patients with SHF stage F2 and healthy controls, and ^§^*P* value < 0.05 for comparisons between patients with SHF stage F4 and and those with SHF stage F2.

^a^SHF was staged from F0 to F4 according the METAVIR scoring system

^b^Student’s t-test for comparisons between patients with SHF stage F2 and healthy controls

^c^Student’s t-test for comparisons between patients with SHF stage F4 and those with SHF stage F2

**Fig. 1:**
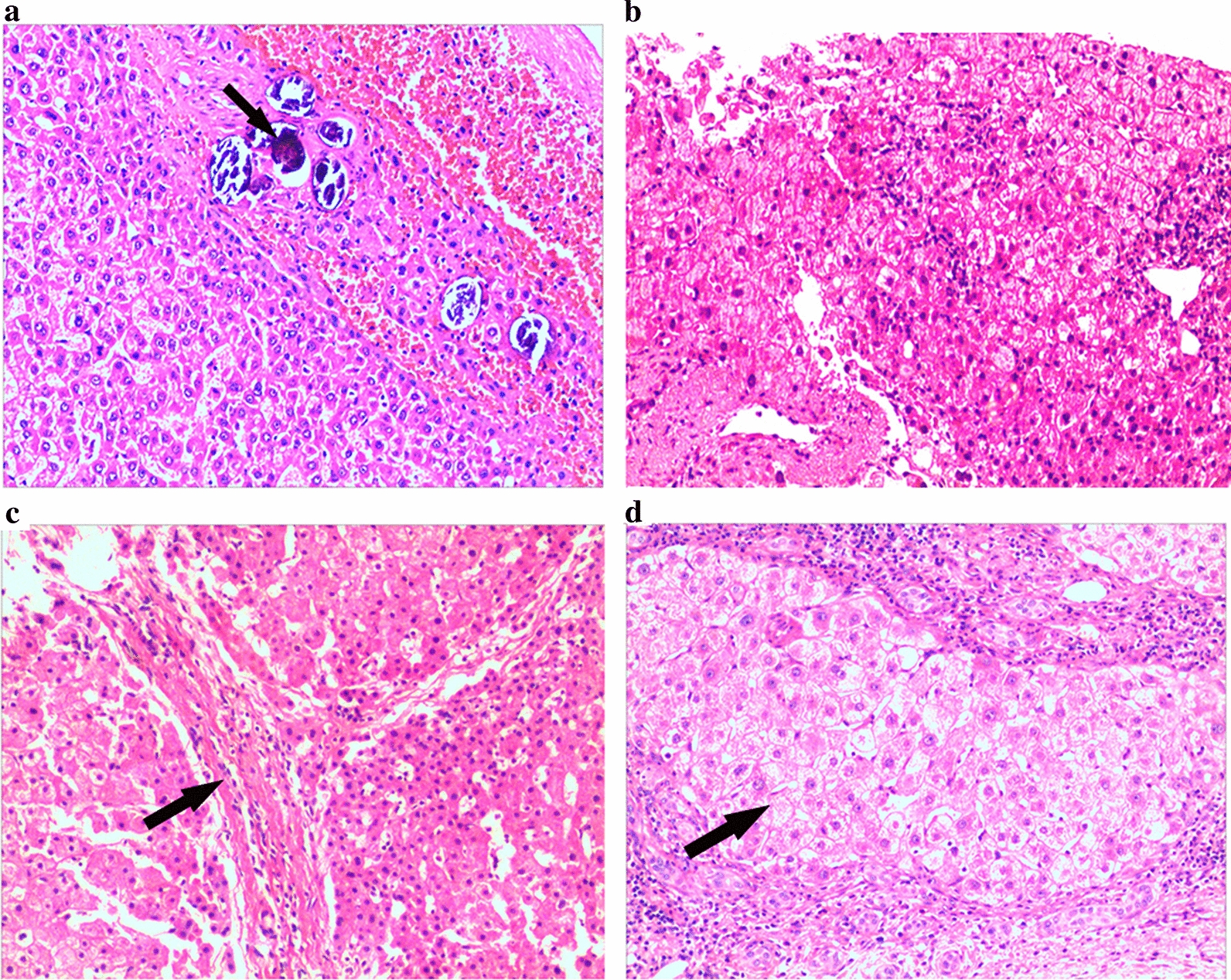
Representative photomicrographs of liver biopsy stained with hematoxylin and eosin (magnification 100×) in patients infected with* Schistosoma japonicum*. **a** Liver tissue sample from a patient with advanced *S. japonicum*-like eggs trapped in liver tissue (black arrow). Histological grading of liver fibrosis was according to the METAVIR scoring system with F0 denoting no fibrosis (**b**), F2 denoting periportal fibrosis (**c**; black arrow) and F4 denoting cirrhosis (**d**; black arrow)

All serum samples were collected and immediately processed according to standard operating procedures to minimize preanalytical variation. The serum samples from the 18 healthy controls and 36 patients with *S. japonicum* infection were divided into three groups: healthy controls (group A, *n* = 18), SHF-F2 group (group B, *n* = 18) and SHF-F4 group (group C, *n* = 18) (Table 1; Fig. 1). For each group of 18 samples, every six individual samples containing equal volumes of serum were mixed, and the pooled serum was then separated into the high- and low-abundance protein fractions on a Human Multiple Affinity Removal System Column (Agilent Technologies, Santa Clara, CA, USA) according to the manufacturer’s instructions. Six replicates of protein extracts per group were used to minimize the individual differences among patients. The use of human serum and biopsy samples in this project was approved by the Institutional Review Board of The Affiliated Hospital of Jiaxing University (LS2021-KY-027). Written consent was obtained from all participants in this study.

### Protein preparation and digestion

A 5-kDa ultrafiltration centrifuge tube (Sartorius, Göttingen, Germany) was used to desalinate and concentrate the components in the high- and low-abundance fractions. The proteins were precipitated with SDT lysis buffer at 95 °C for 15 min. After centrifugation at 14,000 *g* for 20 min, the protein concentration in the supernatant was quantified with a BCA Protein Assay Kit (Bio-Rad, Hercules, CA, USA).

Protein digestion was performed according to the filter-aided sample preparation (FASP) method as described previously [28]. In brief, protein samples (200 μg) for each condition were dissolved in UA buffer (8 M urea, 150 mM Tris–HCl, pH 8.0) and transferred to a new ultrafiltration centrifuge tube by repeated ultrafiltration (Microcon® Centrifugal Filter Unit, 10 kDA; Merck Millipore, Burlington, MA, USA). Then, 100 μl of iodoacetamide (IAA; 100 mM in UA buffer) was added to the protein samples, and the samples were further incubated at room temperature in the dark for 30 min. The filters were then washed three times with 100 μl of UA buffer and two times with 100 μl of 25 mM NH_4_HCO_3_ buffer. For trypsin digestion, the protein suspensions were incubated with 4 μg of trypsin (Promega, Madison, WI, USA) in 40 μl of 25 mM NH_4_HCO_3_ buffer at 37 °C overnight. After trypsin digestion, the resulting peptides were desalted on a C18 cartridge column (Sigma-Aldrich, St. Louis, MO, USA) and vacuum dried; the resulting peptides were desalted on a Waters Oasis C18 solid-phase extraction column (Agilent Technologies, Inc., Santa Clara, CA, USA) and lyophilized for peptide fractionation.

### Data-dependent acquisition- and DIA-based LC–MS/MS

Each fraction was analysed in data-dependent acquisition (DDA) and DIA modes in a Thermo Scientific Q Exactive HF X mass spectrometer (Thermo Fisher Scientific, Waltham, MA, USA) coupled to an Easy nLC 1200 chromatography system (Thermo Fisher Scientific). The parameters for DDA mode were set as follows: full scan range, 300–1800 m/z; MS1 scan resolution, 60,000 at 200 m/z; automatic gain control (AGC), 3e6; maximum injection time (MIT), 25 ms; charge state screening, enabled; and dynamic exclusion, 30 s. Each full MS SIM-mode scan (MS-SIM) followed 20 data-dependent MS2 scans. MS2 scans were performed at a resolution of 15,000, an AGC target of 5e4, a MIT of 250 ms and a normalized collision energy of 30 eV.

The peptides from each sample were analysed by LC–MS/MS in DIA mode. Each DIA cycle contained one full MS-SIM scan and 30 DIA scans covering a mass range of 350–1800 m/z with the following settings: SIM full scan at a resolution of 120,000 at 200 m/z with a MIT of 50 ms and an AGC target of 3e6 in profile mode; followed by DIA scans with a resolution of 15,000 (AGC target, 3e6; MIT, auto; and normalized collision energy, 30 eV).

### MS data analysis

To analyse DDA library data, the FASTA sequence database was searched with Spectronaut software (ver. 14.4.200727.47784; Biognosys AG, Schlieren, Switzerland) against a human database in the UniProt database of protein sequence and annotation data (2 September 2020; 9802 sequences). For analysis, raw data were used as input files, and the corresponding parameters and databases were set for subsequent identification and quantitative analysis. For peptide identification, the searches were run using the following parameters: enzyme, trypsin; maximum number of missed cleavages of 2; dynamic modification, oxidation (M); fixed modification, carbamidomethyl (C); and acetyl of protein, N-term. Additionally, all data were reported based on a 99% confidence level for protein identification as determined by a false discovery rate (FDR) of < 1.0%. A spectral library was constructed using DDA data with Spectronaut Pulsar X (ver. 12.0.20491.4; Biognosys AG).

The raw DIA data were further analysed with Spectronaut software (version 14.4.200727.47784; Biognosys, Switzerland) by searching the above-mentioned spectral library. The main software parameters were set as follows: retention time prediction type, dynamic iRT; and interference on MS2 level correction and cross run normalization, enabled. All results were filtered with the criterion of an FDR threshold of 1% to obtain significant quantitative data.

### Bioinformatic analysis

A protein was considered to be differentially expressed if the fold change in its expression between the serum samples of patients with the different stages of SHF was > 1.5 or < 0.67, with a *P* value < 0.05 as determined by a paired t-test. All differentially expressed proteins were subjected to hierarchical clustering analysis with Java Tree View software (http://jtreeview.sourceforge.net). The classified proteins were subjected to Gene Ontology (GO) analysis using Blast 2 GO software (http://www.blast2go.com/b2ghome) based on functional annotations for biological processes, molecular functions and cellular components. The Kyoto Encyclopedia of Genes and Genomes (KEGG) database (http://geneontology.org/) was used to annotate the protein pathways with the KEGG Automatic Annotation Server (KAAS; https://www.genome.jp/tools/kaas/).

### Validation of the selected dysregulated proteins by enzyme-linked immunosorbent assay

The expression of the selected proteins in serum samples was verified by enzyme-linked immunosorbent assay (ELISA), as previously described. All serum samples were thawed, centrifuged and aliquoted using ELISA kits (Abcam, Cambridge, UK) for selected dysregulated proteins, following the manufacturer’s instructions. Optical density values were read at 450 nm using a microplate reader (model ELx800; BioTek Inc., Winooski, VT, USA), and concentrations were automatically calculated based on the standard curve and dilution factors. Each sample was tested in triplicate. The inter- and intra-assay coefficients of variation were < 5%.

### Statistical analysis

Quantitative data are expressed as the mean ± standard deviation values. The statistical significance of the differences was analysed using Student’s t-test for comparisons between two groups and one-way analysis of variance for comparisons among multiple groups. Statistical analysis was performed using the Statistical Package for the Social Sciences (SPSS) version 22.0 software (SPSS IBM Corp., Armonk, NY, USA). Differences were considered to be statistically significant if the two-tailed* P* value was < 0.05.

## Results

### Clinical characteristics of the study population

A total of 54 serum samples from SHF patients and healthy controls were used in this study (Table 1). Of these 54 individuals, 18 had histological evidence of good health and were denoted the healthy controls, and 36 patients had histological evidence of *S. japonica* infection (Fig. 1a). The 36 patients with *S. japonica* infection were divided into the SHF-F2 group (*n* = 18) (Fig. 1c) and SHF-F4 group (*n* = 18) (Fig. 1d) according to the METAVIR scoring system. There were no differences in age or sex among the three groups. However, compared to the healthy controls and SHF-F2 patients, the SHF-F4 patients had significantly different laboratory results for alanine aminotransferase (ALT), aspartate aminotransferase (AST), gamma-glutamyltranspeptidase (GGT), platelet count (PLT), total bilirubin (TB), alkaline phosphatase (ALP), total bile acid (TBA), albumin (A), AST-to-PLT ratio index (APRI), prothrombin time (PT), activated partial thromboplastin (APTT), international normalized ratio (INR), and fibrinogen content (FIB). Significant correlations were observed between the degree of SHF and blood biochemical parameters.

### Relative quantification of the serum proteome of individuals with advanced *S. japonicum*-induced hepatic fibrosis

**Fig. 2:**
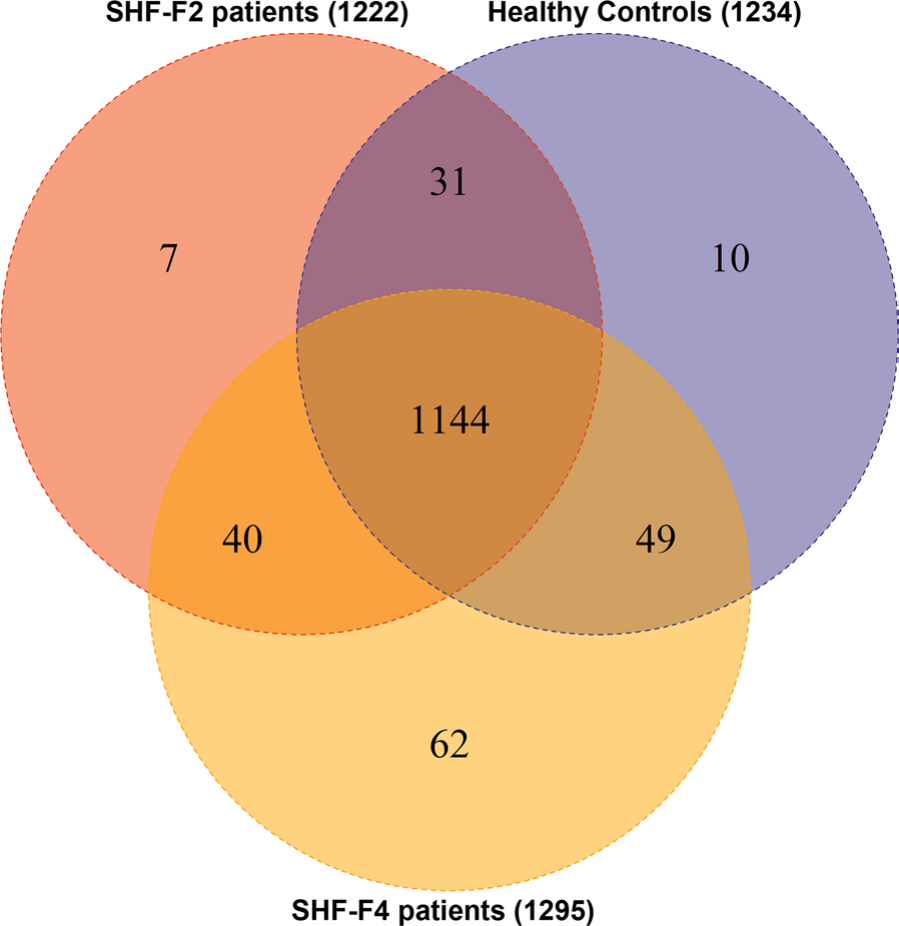
Identification of serum proteins in patients with advanced *S. japonicum-*induced hepatic fibrosis (*SHF-F2*,* SHF-F4*). The Venn diagrams show the numbers of identified proteins and the overlaps of protein identification in three repeated experiments.* SHF-F2*/*SHF-F4* *S. japonicum-*induced hepatic fibrosis stage F2/F4

The DIA-based quantitative proteomic analysis identified 1354 unique proteins across the 9802 peptides (Additional file 1: Tables S1 and S2). Using the results from Spectronaut software (ver. 14.4.200727.47784) with the integrated Andromeda search engine, we quantified 1234, 1222 and 1295 proteins in the three replicates used in DIA-based quantitative proteomic analysis. Among the three replicates, 1144 proteins overlapped, accounting for 82.95% of the total quantified proteins (Fig. 2; Additional file 1: Tables S1 and S2).

### Differentially expressed proteins associated with SHF

**Fig. 3:**
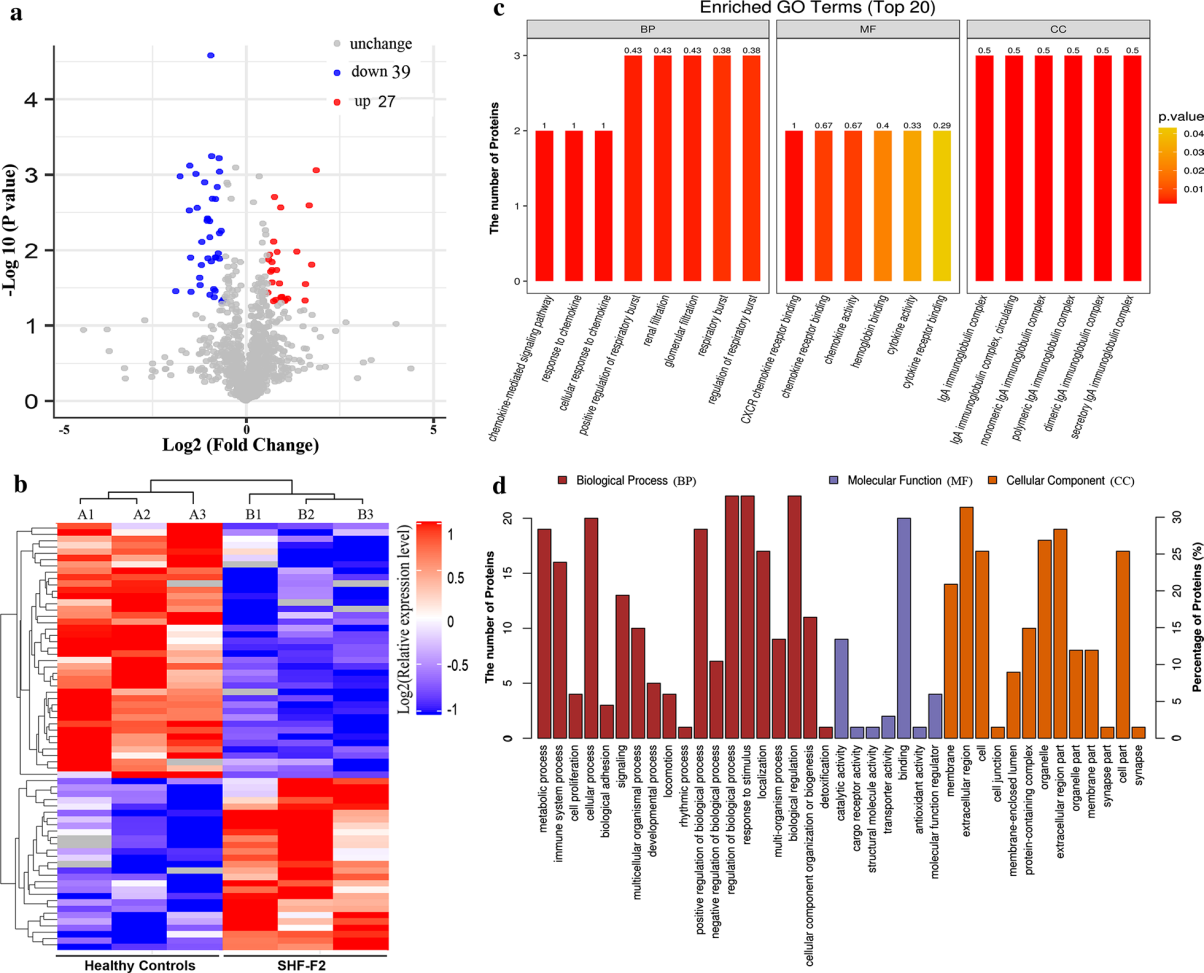
Bioinformatics analysis of differentially expressed proteins in serum samples from healthy controls* vs* those from patients with SHF-F2. **a** Volcano plot representing the changes in protein abundance (groups B [SHF-F2 patients] *vs* A [healthy controls]). A total of 66 dysregulated proteins with a fold change of ≥ ±  1.5 and *P* values < 0.05 were identified. **b** Hierarchical clustering of the 66 dysregulated proteins (groups B* vs* A). **c** Gene ontology (GO) analysis of 66 dysregulated proteins (groups B* vs* A). The abscissa repressents enriched GO function classifications, which are divided into three major categories: biological process (*BP*), molecular function (*MF*) and cellular component (*CC*). **d** Analysis of Eukaryotic Orthologous Groups (KOG) of 66 dysregulated proteins (group B* vs* group A)

To identify the differentially expressed proteins, the relative protein expression values were compared between the study groups (SHF-F2* vs* healthy controls, SHF-F4* vs* SHF-F2). By hierarchical clustering analysis, 66 proteins with a mean expression fold change of ≥ ± 1.5 (log2 = 0.58) were classified as differentially expressed in the serum of SHF-F2 patients compared to the serum of healthy controls (group B *vs* group A, respectively) (Fig. 3a; Additional file 1: Table S1). When the ratio of these 66 proteins was plotted on a heatmap, 27 and 39 proteins were found to be upregulated and downregulated, respectively, between the serum samples from the SHF-F2 and healthy control groups; moreover, these two sets of proteins (upregulated and downregulated, respectively) separated into distinct clusters (Fig. 3b). We then performed GO enrichment analysis to analyse the involvement of the 66 dysregulated proteins in biological processes and found that these differentially expressed proteins separated into distinct clusters. The top three biological process terms in this study were regulation of biological process (*n* = 22), response to stimulus (*n* = 22) and biological regulation (*n* = 22) (Fig. 3c, d). These results clearly indicated that the molecular backgrounds and molecular mechanisms might differ between the SHF-F2 and healthy control groups.

**Fig. 4:**
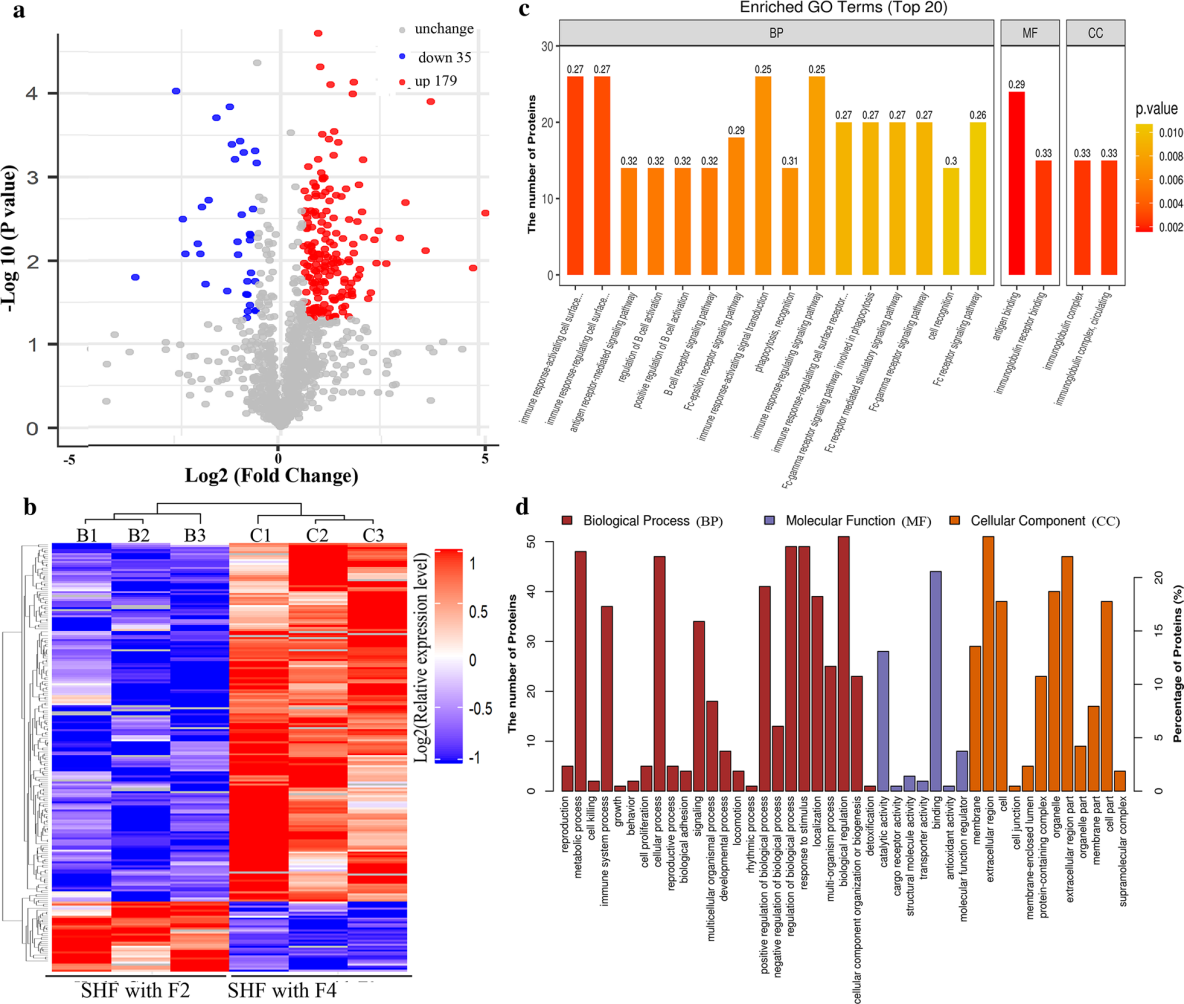
Bioinformatics analysis of differentially expressed proteins in the serum samples from SHF-F2 and SHF-F4 patients. **a** Volcano plot representing the changes in protein abundance (groups C [SFH-F4 patients]* vs* B [SFH-F2 patients]). A total of 214 dysregulated proteins with fold change ≥ ±1.5 and *P* values < 0.05 were identified. **b** Hierarchical clustering of the 214 dysregulated proteins (groups C* vs* B). **c** GO analysis of 214 dysregulated proteins (groups C* vs* B). The abscissa repressents enriched GO function classifications, which are divided into three major ctegories: biological process (*BP*), molecular function (*MF*) and cellular component (*CC*). **d** The 214 dysregulated proteins (groups C* vs* B) were classified in terms of their subcellular localizations

Similarly, serum samples from the SHF-F4 and SHF-F2 groups (group C* vs* group B, respectively) were also compared using the above-mentioned criteria (± log_2_ = 0.58) (Fig. 4a; Additional file 1: Table S2). Figure 4b shows the 214 differentially expressed proteins, of which 179 were upregulated and 35 were downregulated. We further investigated the involvement of these proteins in biological processes by GO enrichment analysis and found that these identified proteins were associated mainly with the terms biological regulation (*n* = 51), regulation of biological process (*n* = 47) and response to stimulus (*n* = 47) (Fig. 4c, d). Furthermore, GO enrichment analysis revealed that these 214 proteins were involved in the molecular functions binding (*n* = 44), protein binding (*n* = 33), and catalytic activity (*n* = 28). We also classified these 214 differentially expressed proteins according to their subcellular localization, and each protein was assigned to at least one term. More than 23% of the proteins were annotated as belonging to the extracellular region, and the other two main cellular components terms associated with these proteins were the extracellular space (21.96%) and organelle (18.69%) (Fig. 4d).

### KEGG pathway analysis of the differentially expressed proteins

**Fig. 5:**
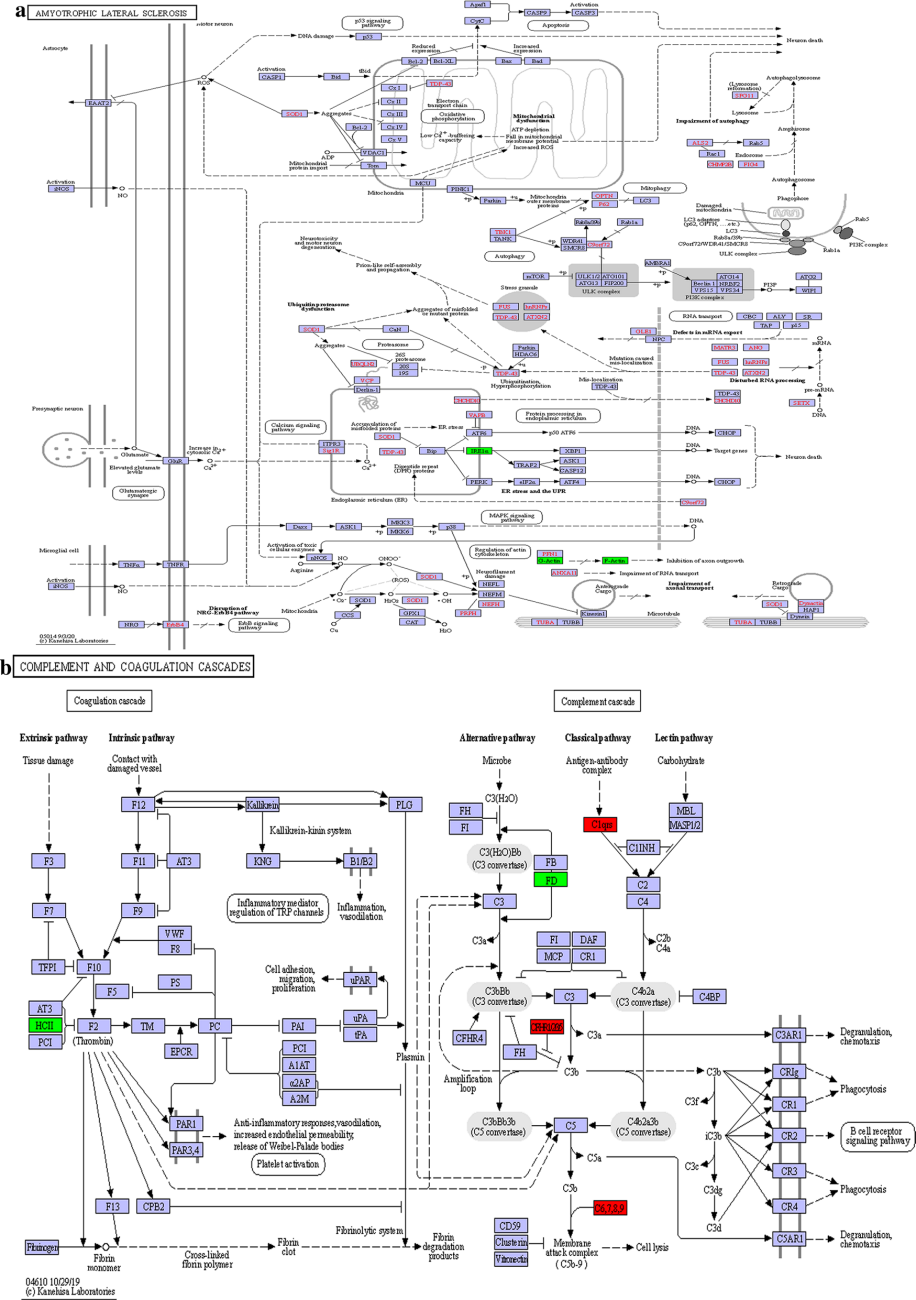
The key signaling pathways involved in the advanced *S. japonicum-*induced hepatic fibrosis patient serum samples.** a**,** b** Amyotrophic lateral sclerosis (**a**) and complement and coagulation cascades (**b**) obtained from KEGG pathway-based enrichment analysis of dysregulated proteins

To further analyse the roles of the proteins with SHF-associated alterations in expression, the differentially expressed proteins were blasted against the online KEGG database to retrieve their KEGG orthology identification codes and subsequently mapped to the reference KEGG pathways. As shown in Additional file 1: Table S3; Fig. 5a, the dysregulated proteins between the SHF-F2 and healthy control groups (Groups B* vs* A) participated mainly in the regulation of amyotrophic lateral sclerosis, cytokine–cytokine receptor interaction, viral protein interaction with cytokine and cytokine receptor, complement and coagulation cascades, chemokine signalling pathway and apoptosis pathways, which were the top six modulated signalling pathways enriched with dysregulated proteins. KEGG pathway analysis of proteins with altered expression in blood serum between the SHF-F4 and SHF-F2 groups revealed that the dysregulated proteins were enriched mainly in the complement and coagulation cascades, prion disease, *Staphylococcus aureus* infection and systemic lupus erythematosus pathways (Additional file 1: Table S4; Fig. 5b). Thus, the analysis results also suggest that specific signalling pathways are indeed differentially regulated in patients with advanced *S. japonicum-*induced hepatic fibrosis, although common signalling pathways are also differentially regulated. Therefore, advanced *S. japonicum-*induced hepatic fibrosis can be expected to have its own specific molecular characteristics and molecular mechanisms.

### Verification of the selected differential expression of C1QA and CFD

Based on the hierarchal clustering analysis results (Fig. 3) and key modulated signalling pathways (Fig. 5), the proteins complement C1q subcomponent subunit A (C1QA, 1.82-fold) and complement factor D (CFD, 2.51-fold) were differentially expressed in the SHF-F4 and SHF-F2 groups of schistosomiasis patients. C1QA, one of the three components of the C1q molecule based on the hexamer of trimers composed of three distinct polypeptide chains, is considered to be the first component in the classical pathway and plays a crucial role in the innate immune response, defence against invading pathogens and activation of complement by binding to immune complexes [32, 33]. CFD, a member of the trypsin family, activates the alternative pathway by cleaving C3b-bound factor B and is involved in innate and adaptive defences against pathogen infection [34, 35]. Therefore, these factors may be biomarkers of interest for distinguishing the F2 and F4 stages of advanced *S. japonicum-*induced hepatic fibrosis. Hence, the expression changes in C1QA and CFD were further verified by ELISA using independent sets of 108 serum samples (36 healthy controls, 36 patients with SHF-F2 and 36 patients with SHF-F4).

**Fig. 6:**
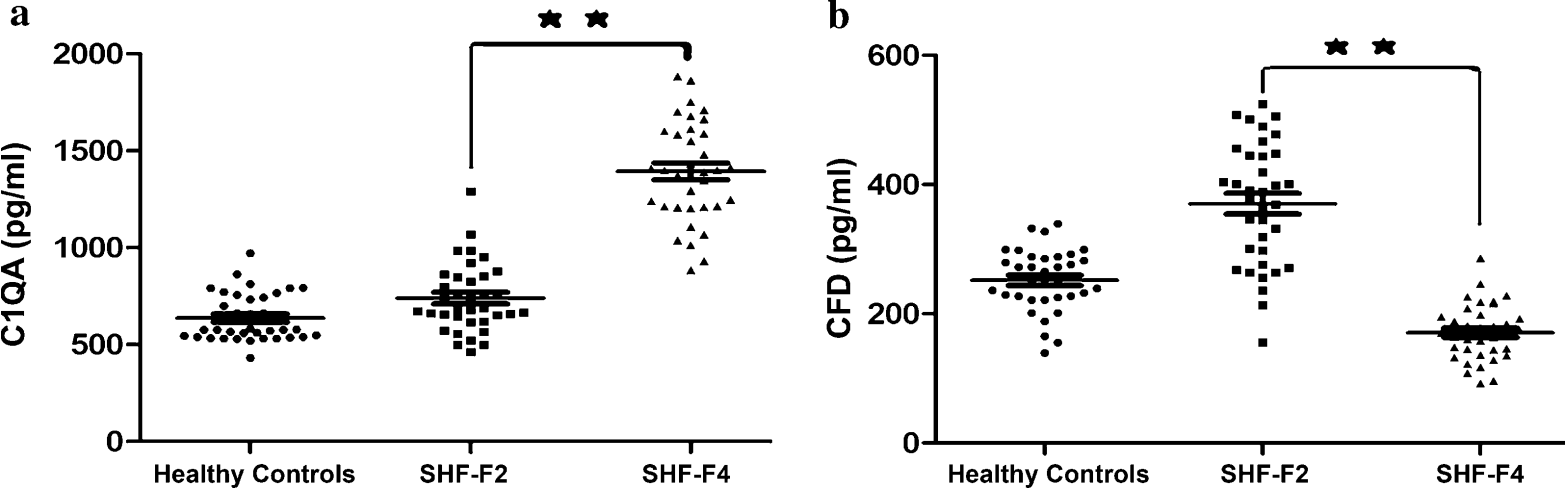
Validation of the selected differential expression proteins of C1QA (**a**) and CFD (**b**) between the SHF-F2 and SHF-F4 schistosomiasis patient serum samples by ELISA in the validation cohort. Asteriks indicate a significant difference at **P < 0.01

Figure 6 shows that the protein expression level of C1QA was significantly upregulated and that of CFD was downregulated in serum samples from schistosomiasis patients in the SHF-F4 group compared to those from patients in the SHF-F2 group. The protein expression of C1QA was significantly upregulated by 2.06-fold (*n* = 36 patients, *P* < 0.01), while the protein expression of CFD was downregulated 2.41-fold (*n* = 36 patients, *P* < 0.01) in SHF-F4 patient serum samples compared to the SHF-F2 patient serum samples.

In summary, we were successful in identifying the *S. japonicum*-induced hepatic fibrosis-associated proteins C1QA and CFD in schistosomiasis patients, consistent with the DIA-based quantitative proteomic analysis results shown in Fig. 3 and Additional file 1: Table S2. Therefore, these two proteins have the potential to be biomarkers for distinguishing between SHF patients, but their underlying molecular mechanisms need further investigation.

## Discussion

Although the incidence of human schistosomiasis is gradually decreasing, this disease is still and will continue to be a major public health concern worldwide due to the associated chronic morbidity and premature death [1, 2]. Schistosomiasis patients with late chronic infection account for a significant proportion of fatal hepatic fibrosis cases, and the results of previous studies confirm that SHF is associated with an increased risk of developing cirrhosis and even HCC compared to that of healthy individuals [12, 36–39]. While much of the existing literature has focused on noting the presence of disparities among patients with schistosomiasis at different stages of SHF, little is known about the potential mechanisms and the differences in specific biological pathways in the context of hepatic schistosomiasis. The serum proteomic data presented in this study are the first to show molecular differences at the proteome level among schistosomiasis patients at different stages of SHF. These results not only confirm those of earlier studies, demonstrating that the degree of SHF is the main determinant of prognosis and influences treatment decisions, but also provide new information regarding hepatic schistosomiasis patients, which will facilitate further in-depth investigation.

A systematic approach for investigating changes in the serum proteome is essential to a comprehensive understanding of the complex pathogenesis of SHF. In the present study, we applied DIA–MS-based quantitative proteomic analysis of serum samples from three groups—healthy control individuals, patients with SHF-F2 and patients with SHF-F4—to quantify the dynamic changes in the global serum proteome of schistosomiasis patients at different stages of SHF. Significant and large differences were found among the healthy control, SHF-F2 and SHF-F4 groups at the molecular level through comprehensive analysis of the differentially expressed proteins. Among the 1343 proteins with FDR < 1% identified in our study, 82.95% were common to all three biological replicates, indicating the stability of the work flow and the reliability of the conclusion drawn. Based on the identification criteria for dysregulated proteins (fold change ≥ ± 1.5, *P* < 0.05), 66 differentially expressed proteins between the healthy control and SHF-F2 groups and 214 differentially expressed proteins between the SHF-F2 and SHF-F4 groups were identified in the serum samples. Although all of the signalling pathways associated with the above-mentioned dysregulated proteins are key players in the development of SHF, only the complement and coagulation cascades signalling pathway was extensively involved in both comparison groups (healthy control* vs* SHF-F2, SHF-F2* vs* SHF-F4) of hepatic schistosomiasis patients. This finding demonstrates that in patients with advanced *S. japonicum-*induced SHF, the complement and coagulation cascades signalling pathway may contribute to the regulation of hepatic fibrosis and liver cirrhosis caused by excessive extracellular matrix deposition by regulating the expression of these differentially expressed proteins.

The complement and coagulation cascades, which function as major proteolytic cascades in blood serum, are reported to be composed of more than 35 soluble plasma proteins, and recent evidence suggests that the complement and coagulation cascades mediate the response to acute injury by limiting bleeding and pathogen invasion and facilitating wound healing [33, 40–42]. Additionally, several studies have shown that the complement and coagulation cascades are not merely processes reactive to inflammation, immunity and haemostasis but also rather critical determinants of liver disease pathogenesis [34, 37]. Here, our data support the hypothesis that the effects of the complement and coagulation cascades on the SHF stage strongly depend on the degree of complement and coagulation cascade signalling pathway activation or inhibition. Therefore, unsurprisingly, the complement and coagulation cascades signalling pathway was involved in both groups.

The expression of C1QA was significantly upregulated in the serum samples of SHF-F4 patients compared to those of healthy control individuals and SHF-F2 patients. C1QA is the main recognition protein in the classical complement pathway and plays key roles in the innate immune response and defence against dangerous factors in the presence of injured cells, accumulating debris or foreign materials [32, 33]. On the other hand, it is associated with several modulatory processes, including pathogen clearance, angiogenesis, autoimmunity, apoptosis, tolerance, chemotaxis and homeostasis. Consistent with this function, upregulation of C1QA expression in the complement cascade can result in increased susceptibility to complement-mediated liver damage and fibrosis [43, 44]. The results of this study indicate that upregulation of C1QA expression may increase transcriptional activation of the classical complement pathway* via* the formation of C3 and C5 convertases in SHF-F4 patient serum samples compared with SHF-F2 patient and healthy control serum samples. The activated complement pathway may play a inhibitory role in hepatic fibrosis through activation of the C1 complex (C1qrs) triggered by antigen–antibody complexes, because complement pathway activation is required for the upregulation of many proteins, such as C1QA. Thus, we discovered and validated that the expression level of C1QA in serum samples from patients with advanced *S. japonicum*-induced hepatic fibrosis was a positive marker for distinguishing the stage of SHF.

The expression of CFD was also significantly downregulated in the serum of SHF-F4 patients compared to the serum of SHF-F2 patients. CFD is the enzyme involved in the rate-limiting step of the alternative complement pathway, namely the cleavage of factor B, the larger fragment of which remains bound to complement C3b to form the alternative pathway C3(H2O)Bb (C3 convertase–C3bBb) [34, 35, 44]. Consistent with the KEGG pathway analysis results suggesting that the complement pathway plays an important role in inflammation, phagocytosis, antibody production, clearance of foreign cells and molecules and killing of susceptible cells, many proteins associated with those functions, including CFD, were dysregulated in our data. In addition, consistent with these collective findings, our observations indicate that the expression of CFD and other soluble regulators of the alternative pathway, including factor H and C4bp, is reduced in SHF-F4 patients. These studies provide only associative evidence for a role of the alternative pathway in advanced *S. japonicum-*induced hepatic fibrosis. However, the underlying mechanism by which CFD inhibits the alternative pathway remains unclear. Therefore, the expression of CFD in the serum samples from SHF-F2 and SHF-F4 patients may be a potential biomarker for distinguishing the stage of advanced *S. japonicum-*induced hepatic fibrosis.

Overall, we profiled serum proteins from healthy controls and from SHF-F2 and SHF-F4 patients and identified two potential biomarkers for distinguishing the stage of advanced *S. japonicum*-induced hepatic fibrosis.

## Conclusions

In this study, we applied DIA-based MS analysis to study the alterations in the serum proteome profile among healthy controls, SHF-F2 patients and SHF-F4 patients. As expected, our results clearly prove that different protein profiles and signalling pathways are involved in advanced *S. japonicum-*induced hepatic fibrosis. To verify these results, the differences in the expression levels of C1QA and CFD in samples from patients with advanced *S. japonicum-*induced hepaticfibrosis were investigated by ELISA. In addition, the results of the present study suggest the potential application of the C1QA and CFD proteins as biomarkers for distinguishing between individuals with SHF-F2 and SHF-F4 (Additional file 2).

## Supplementary Information

**Additional file 1: Table S1.** Differentially expressed proteins of serum samples between SHF with F2 and healthy controls (sheet 1). **Table S2.** Differentially expressed proteins of serum samples between SHF with F4 and SHF with F2 (sheet 2). **Table S3.** KEGG pathway analysis of the differentially expressed proteins of serum samples between SHF with F2 and healthy controls (fold change of ≥  ± 1.5) (sheet 3). **Table S4.** KEGG pathway analysis of the differentially expressed proteins of serum samples between SHF with F4 and SHF with F2 (fold change of ≥  ± 1.5) (sheet 4).

**Additional file 2: Figure S1.** Overall technical route of this project. **Figure S2.** The qualities of the proteome dataset.** A** Quantitative heatmap of DIA.** B** The scores of principle component analysis (PCA).** C** Chart of iRT elution time.** D** Column peak capacity statistics.** E** Protein scores of FDR.** F** The scatterplot of QC. **Figure S3.** Distribution analysis of differentially expressed proteins.** A** The protein ratio distribution between the healthy control individual and SHF-F2 patient serum samples.** B** The protein ratio distribution between SHF-F4 and SHF-F2 patient serum samples.

## Data Availability

All data generated during this study are included in this published article. A total list of identified proteins has been uploaded as additional files. The mass spectrometry proteomics data have been deposited in the ProteomeXchange database (http://www.proteomexchange.org) with px-submission number 492678.

## Abbreviations

C1QA: Complement C1q subcomponent subunit A
CFD: Complement factor D
DDA: Data-dependent acquisition
DIA: Data-independent acquisition
ELISA: Enzyme-linked immunosorbent assay
FASP: Filter-aided sample preparation
GO: Gene ontology
KEGG: Kyoto Encyclopedia of Genes and Genomes
SHF: Schistosomiasis hepatic fibrosis
