# Supplementary material for: Serum proteomic profiling in patients with advanced Schistosoma japonicum-induced hepatic fibrosis
Source: Parasit Vectors. 2021 May 1;14:232. doi: 10.1186/s13071-021-04734-1 (PMC8088642; doi:10.1186/s13071-021-04734-1)
Supplement: Supplementary file 2 — Additional file 2: Figure S1. Overall technical route of this project. Figure S2. The qualities of the proteome dataset. A Quantitative heatmap of DIA. B The scores of principle component analysis (PCA). C Chart of iRT elution time. D Column peak capacity statistics. E Protein scores of FDR. F The scatterplot of QC. Figure S3. Distribution analysis of differentially expressed proteins. A The protein ratio distribution between the healthy control individual and SHF-F2 patient serum samples. B The protein ratio distribution between SHF-F4 and SHF-F2 patient serum samples. [file 13071_2021_4734_MOESM2_ESM.docx]

**Additional file 2**

Serum proteomic profiling in patients with advanced *Schistosoma japonicum-*induced hepatic fibrosis

Jing Huang^1,2,3^，Xinguang Yin^4^，Lifang Zhang^1,2^，Ming Yao ^1,2^, Dahai Wei^1,2,^* and Yiming Wu^1,2,^*

^1^Institute of Hepatology, The Affiliated Hospital of Jiaxing University, Jiaxing, Zhejiang Province 314001, PR China.

^2^Institute of Hepatology, The First Hospital of Jiaxing, Jiaxing, Zhejiang Province 314001, PR China.

^3^Department of Clinical Medicine, Bengbu Medical College, Bengbu, Anhui Province 233030, PR China.

^4^Jiaxing Maternity and Child Health Care Hospital, Jiaxing, Zhejiang Province 314001, PR China.

* Correspondence: weidahai3166@hotmail.com, jxyywxb@163.com

E-mails:

JH: huangjinging2020@163.com

XY: jxyyyxg@sina.com

LZ: 1191844658@qq.com

MY: jxyaoming@163.com

DW: weidahai3166@hotmail.com

YW: jxyywxb@163.com

**Supplementary Figures:**


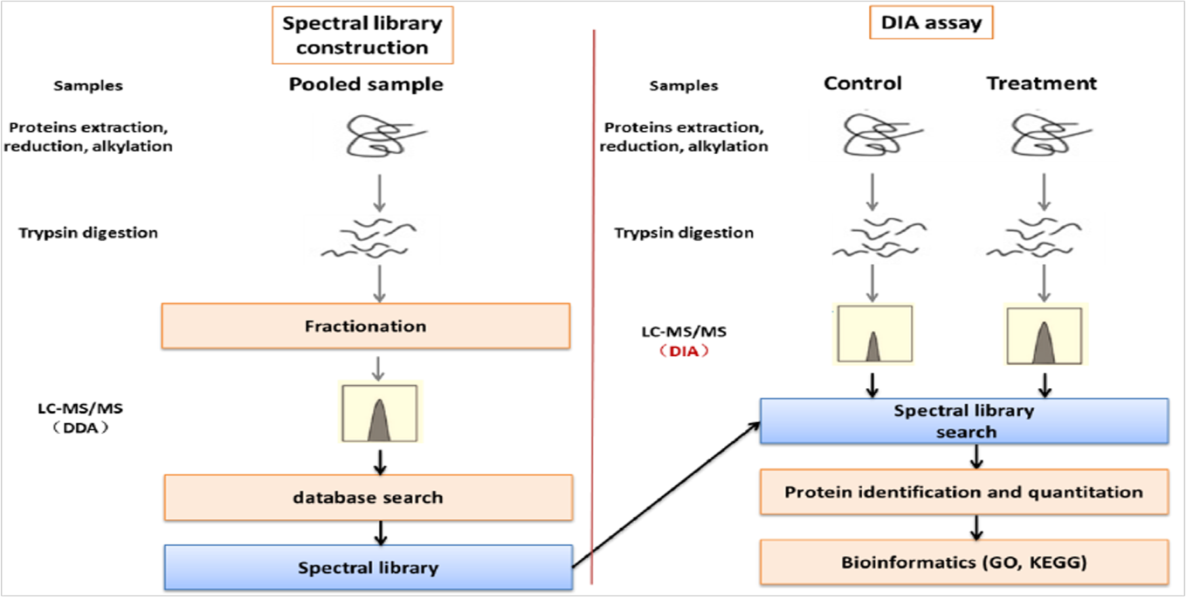


Figure S1. Overall technical route of this project.


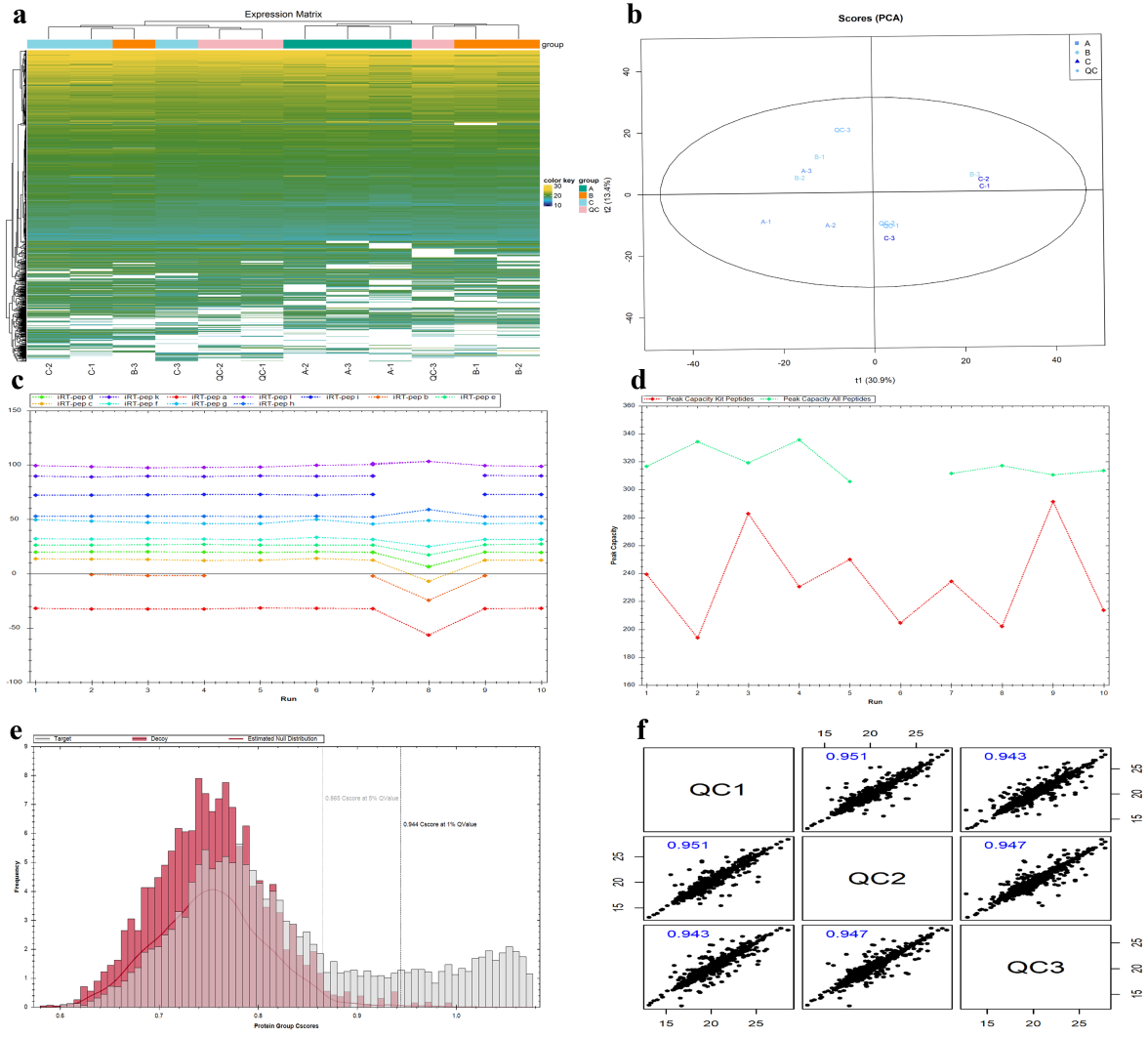


Figure S2. The qualities of the proteome dataset. (a) Quantitative heatmap of DIA. (b) The scores of Principle Component Ananlysis (PCA). (c) Chart of iRT elution time. (d) Column peak capacity statistics. (e) Protein scores of FDR. (f) The scatterplot of QC.


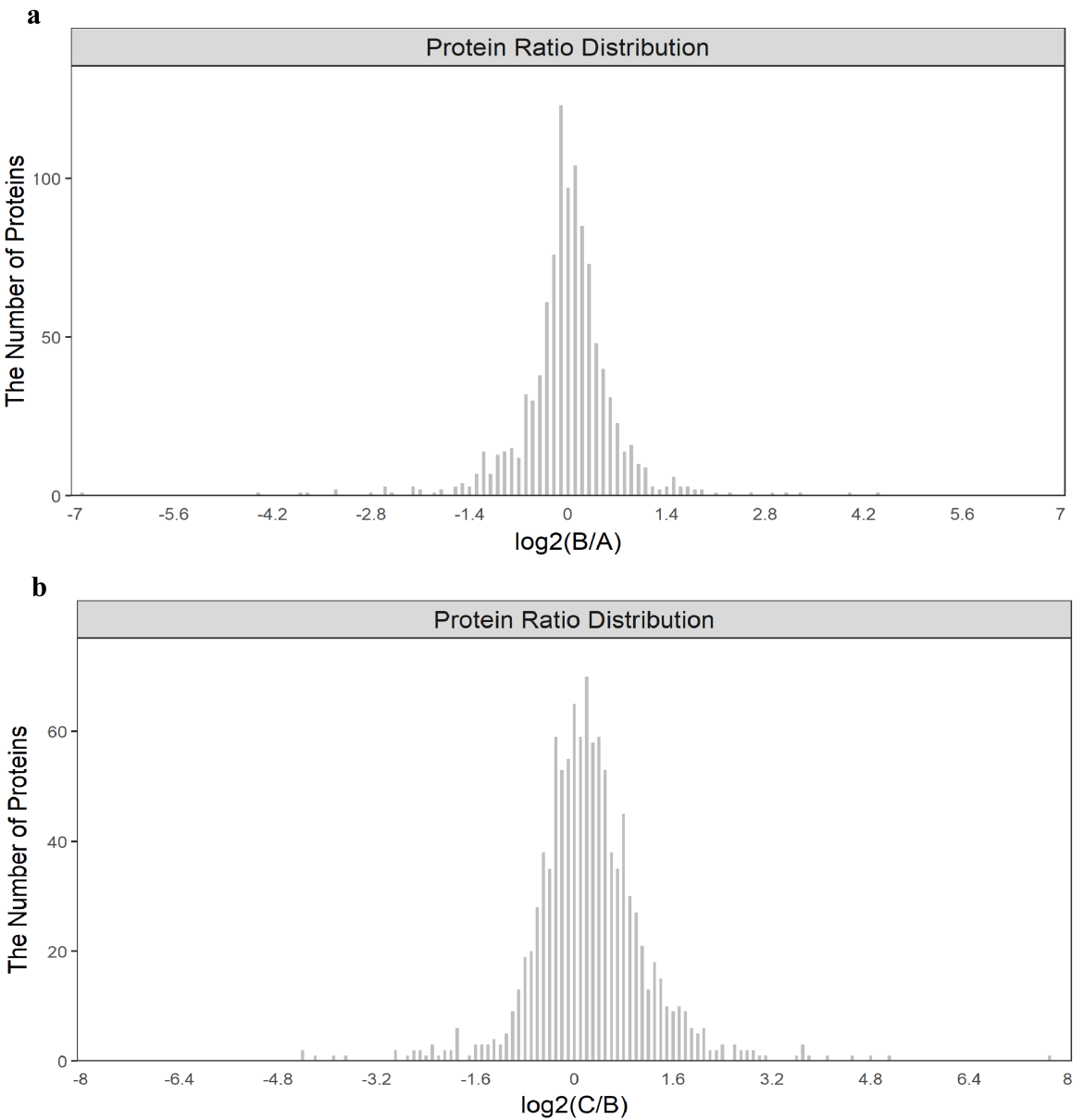


Figure S3. Distribution analysis of differentially expressed proteins. (a) The protein ratio distribution between the healthy control individual and SHF-F2 patient serum samples. (b) The protein ratio distribution between SHF-F4 and SHF-F2 patient serum samples.
